# A candidate effector protein PstCFEM1 contributes to virulence of stripe rust fungus and impairs wheat immunity

**DOI:** 10.1007/s44154-022-00042-5

**Published:** 2022-04-08

**Authors:** Xingxuan Bai, Huan Peng, Farhan Goher, Md Ashraful Islam, Sanding Xu, Jia Guo, Zhensheng Kang, Jun Guo

**Affiliations:** State Key Laboratory of Crop Stress Biology for Arid Areas, College of Plant Protection, Northwest A&F University, Yangling, 712100 Shaanxi China

**Keywords:** *Pst*, CFEM domain, Effector, Virulence, Wheat

## Abstract

Common in Fungal Extracellular Membrane (CFEM) domain proteins are considered to be unique to fungi and closely related to pathogenicity. However, the *Puccinia striiformis* f. sp. *tritici* (*Pst*) effector containing the CFEM domain has not been reported. Here, we obtained an effector, PstCFEM1, containing a functional N-terminal signal peptide sequence and the CFEM domain from *Pst* race CYR31. qRT-PCR assay indicated that the transcript levels of *PstCFEM1* were highly induced during the early stages of infection. Overexpression of *PstCFEM1* suppressed Pst322 (an elicitor-like protein of *Pst*)-trigged cell death, reactive oxygen species (ROS) accumulation and callose deposition. Host-induced gene silencing (HIGS) experiments showed that knockdown of *PstCFEM1* decreased the virulence of *Pst*, while ROS accumulation in silenced plants increased near the infection site. In addition, wheat containing the *PstCFEM1*-silenced construct increased resistance to multiple races of *Pst*. Our data suggest that *PstCFEM1* suppresses wheat defense by inhibiting ROS accumulation and contributes to increased virulence of *Pst*.

**Supplementary Information:**

The online version contains supplementary material available at 10.1007/s44154-022-00042-5.

## Introduction

Wheat stripe rust, caused by the basidiomycetous fungus *Puccinia striiformis* f. sp. *tritici* (*Pst*), one of the most common and destructive wheat diseases in most wheat growing areas (Wellings, 2011; Chen et al., 2014), has been one of the most significant biotic threats to global wheat production in the twenty-first century (Schwessinger, 2017). Present strategies to manage this disease depend on resistant cultivars combined with fungicide application (Chen et al., 2014). Hence, new viable methods must be discovered to protect wheat crops from rust fungi (Fisher et al., 2012). Understanding the molecular basis of *Pst* pathogenesis and the *Pst*–wheat interaction will contribute new strategies for long-term stripe rust control (Brown, 2015; Chen et al., 2014).

*Pst* is an obligate biotrophic parasite that cannot be cultured in vitro (Staples, 2000) and can only invade living host tissues, where it forms haustoria that invade the host cells to absorb nutrients (Voegele & Mendgen, 2011). Because *Pst* lacks a reliable and efficient transformation mechanism, the function of only a few effectors has been studied (Petre et al., 2014). The use of the host-induced gene silencing (HIGS) technique has greatly aided the study of *Pst* effectors and research related to pathogenicity (Yin & Hulbert, 2015; Qi et al., 2019b). Pst18363, a stripe rust effector, targets and stabilizes TaNUDX23 that encourages stripe rust disease (Yang et al., 2020). To overcome reactive oxygen species (ROS)-induced defense in wheat, *Pst* effector PstGSRE1 interrupts nuclear localization of ROS-promoting transcription factor TaLOL2 (Qi et al., 2019a). An effector protein Pst_12806 of *Pst* targets the wheat TaISP protein (a putative component of the cytochrome b6-f complex) and suppresses chloroplast function (Xu et al., 2019). Pst_8713 is an effector that impairs plant immunity and enhances *Pst* virulence (Zhao et al., 2018). Effector PEC6 overwhelms pattern-triggered immunity (PTI) in a host species-independent manner (Liu et al., 2016). However, there have been no reports indicating that silencing of effector gene confers broad-spectrum resistance of wheat against *Pst*.

Fungal extracellular membrane proteins commonly include the CFEM domain (Zhang et al., 2015), a protein domain unique to fungi, containing distinctive eight cysteine residues (Kulkarni et al., 2003). Many studies have shown that the CFEM protein is closely linked to fungal pathogenicity. CFEM domain protein MoPTH11 of *Magnaporthe oryzae* functions as an intracellular signal molecule in appressoria and bud tracheids which is required for proper development of the appressoria, and appressoria-like structures (Kou et al., 2017). CFEM domain protein MoACI1 directly affects the development of appressoria (Kulkarni & Dean, 2004; Salomon et al., 2014). CFEM domain protein MoWISH is essential for surface sensing, asexual and pathogenic differentiation in *M. oryzae* (Sabnam & Barma, 2017). CFEM domain proteins CSA1 of *Candida albicans* and AG2 of *Coccidioides immitis* regulate the growth of hyphae and pathogenicity (Peng et al., 1999; Lamarre et al., 2000). Pathogenicity, conidial development, and stress tolerance are all influenced by BcCFEM1 in *Botrytis cinerea* (Zhu et al., 2017). However, an effector containing the CFEM domain in the stripe rust fungus has not been documented.

In the current study, we isolated and characterized a candidate effector PstCFEM1 from *Pst* and used overexpression and HIGS technologies to explore its function. Our results indicated that PstCFEM1 facilitates *Pst* infection by suppressing ROS accumulation and wheat plants containing the *PstCFEM1*-silenced construct show broad-spectrum resistance to *Pst*.

## Results

### Identification of the candidate effector

A number of *Pst* secreted proteins have been identified (Zheng et al., 2013). In our previous study using an *A. tumefaciens*-mediated transient expression assay in *N. benthamiana*, 30 effectors were selected by *Agrobacterium* carrying mouse pro-apoptotic protein-bax (Yang et al., 2020). A candidate effector PstCFEM1 was shown to suppress bax-triggered programmed cell death (PCD) (Fig. S1). *PstCFEM1* was cloned from *Pst* race CYR31 and found to have an open reading frame of 579 bp, encoding a protein of 192 amino acids. The sequence of this protein contains a CFEM domain and is consistent with the sequence of hypothetical protein PSTG_04849 through NCBI Blastp (GenBank accession number KNF02028.1). Since there is no report of this type of effector in *Pst*, we named this protein “PstCFEM1” as this is the first report of a CFEM protein in *Pst*.

### *PstCFEM1* is significantly induced by *Pst* infection

**Fig. 1:**
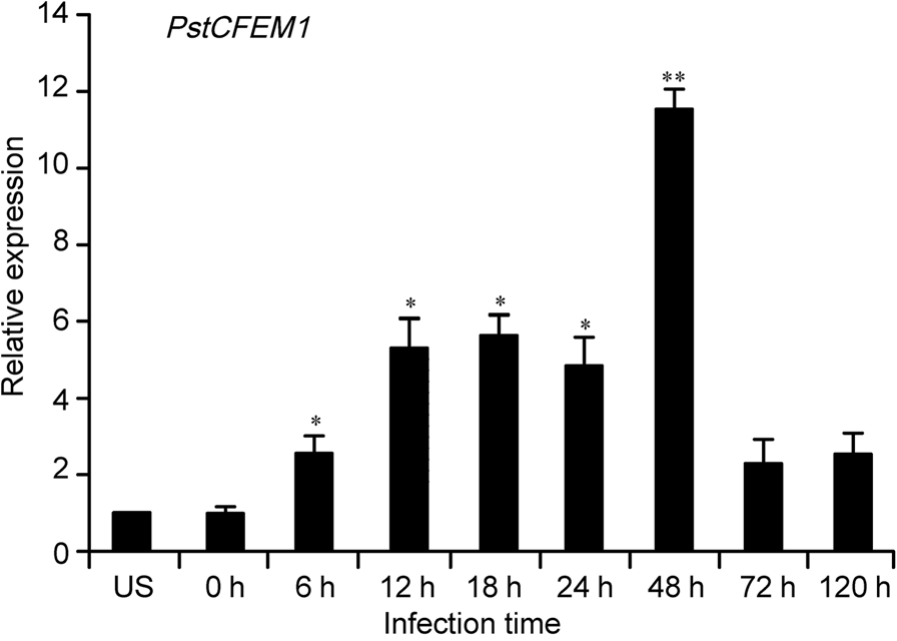
Transcript profiles of *PstCFEM1* in different *Pst* infection stages. Wheat leaves inoculated with compatible race CYR31 were sampled at 0, 6, 12, 18, 24, 48, 72 and 120 hpi. Error bars represent the variations among three independent replicates. qRT-PCR values were normalized using *TaEF-1α*. Differences between time-course points were assessed using student’s *t*-test (*, *P* < 0.05; **, *P* < 0.01)

To determine whether *PstCFEM1* is involved in *Pst* infection, a qRT-PCR assay was used to analyze *PstCFEM1* transcript levels at different *Pst* infection stages. The transcript levels were gradually induced as early as 6 h post inoculation (hpi) and attained the maximum level of 11.5-fold at 48 hpi compared with the control. Then, the transcript level returned to the original level at 72 hpi (Fig. 1). This result indicates that transcription of *PstCFEM1* is induced by *Pst* infection.

### Secretion validation of the N-terminal signal peptide of PstCFEM1

**Fig. 2:**
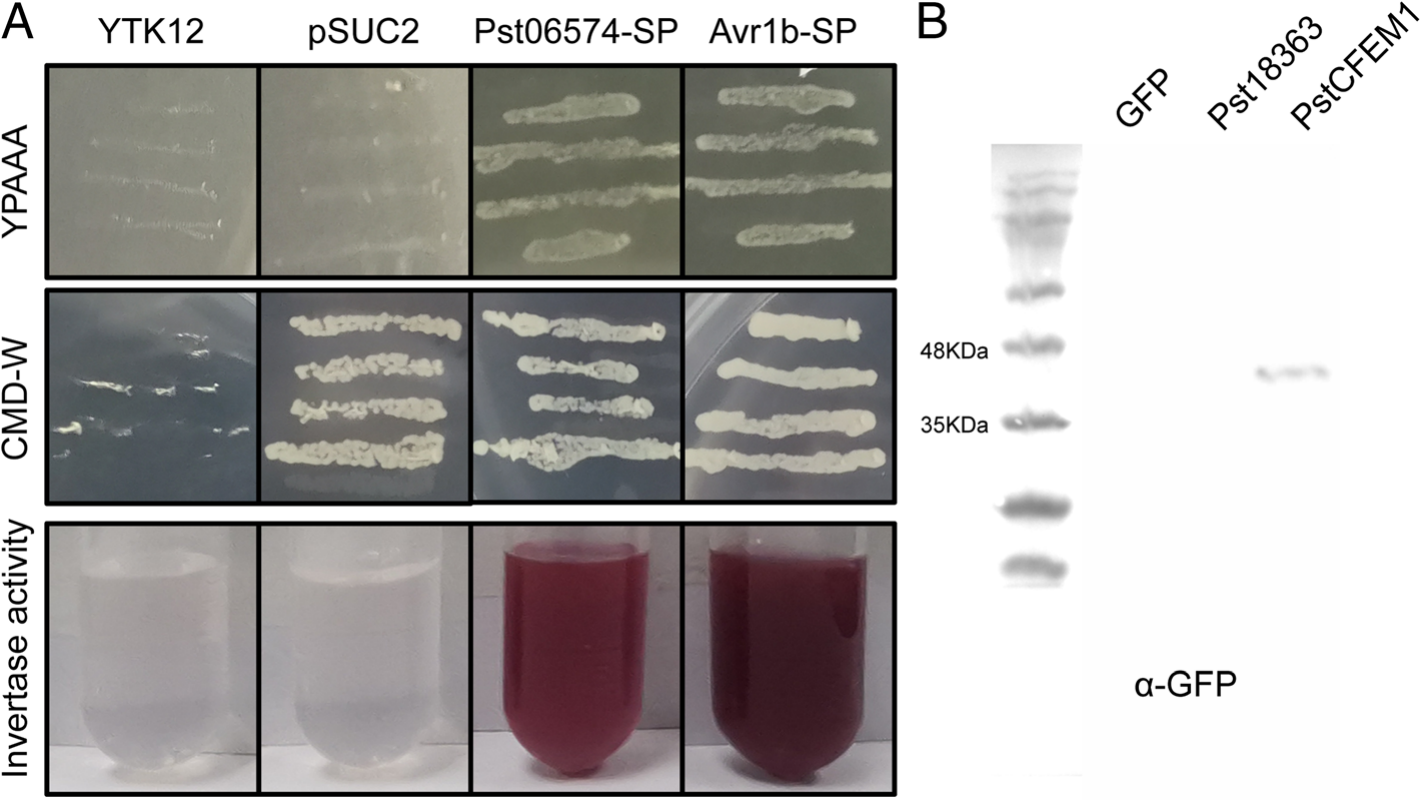
Functional validation of the signal peptide of PstCFEM1. **A** The sequence of the putative PstCFEM1 signal peptide was fused in-frame to the invertase sequence in the pSUC2 vector and transformed into yeast strain YTK12. The untransformed YTK12 strain and empty pSUC2 vector were used as negative controls, and the oomycete effector Avr1b from *Phytophthora sojae* was used as a positive control. Only yeast strains that are able to secrete invertase can grow on YPRAA media. TTC is reduced to red formazan when invertase is secreted. **B** Western blot of PstCFEM1 in intercellular fluid. Cytoplasmic effector Pst18363 from *Pst* as negative control. Three independent biological replicates were conducted for each experiment

SignalP 5.0 predicted that PstCFEM1 has a signal peptide encoded by the first 75 bp (Fig. S2). To confirm the secretory function of the predicted signal peptide of PstCFEM1, we used a signal sequence trap system (Zhao et al., 2018). Empty vector pSUC2 was used as a negative control, and the signal peptide of effector Avr1b from *Phytophthora sojae* was used as a positive control (Gu et al., 2011). Similar to signal peptide of Avr1b, the signal peptide of PstCFEM1 enabled the invertase mutant yeast strain to grow on CMD-W medium (yeast growth without invertase secretion) and YPRAA medium (yeast growth only when invertase is secreted) (Fig. 2). In addition, to further confirm the secretory function of the signal peptide, we tested invertase enzymatic activity, in which secreted invertase reduced TTC to insoluble red colored TPF. We found that the TTC-treated signal peptides of PstCFEM1 and positive control culture filtrates turned red, whereas the negative control culture filtrates treated with TTC remained colorless (Fig. 2). This result verified the secretory function of N-terminal signal peptide of PstCFEM1.

To further investigate its localization, we firstly performed bioinformatic analysis. The results indicated that PstCFEM1 has no predicted transmembrane region (Fig. S3) but has a predicted GPI-anchor site (Fig. S4), and it’s a putative apoplastic effector (Fig. S5). To verify the prediction, PstCFEM1 protein in the intercellular fluid of *N. benthamiana* was detected using the western blot method. As shown in Fig. 2B, PstCFEM1 was present in the intercellular fluid, but the GFP protein was not detected. In addition, Pst18363 which had been identified as a cytoplasmic effector (Yang et al., 2020) was not been detected in the intercellular fluid (Fig. 2B). Thus, the results suggest that PstCFEM1 is an apoplastic effector.

### PstCFEM1 suppresses PCD and callose deposition

**Fig. 3:**
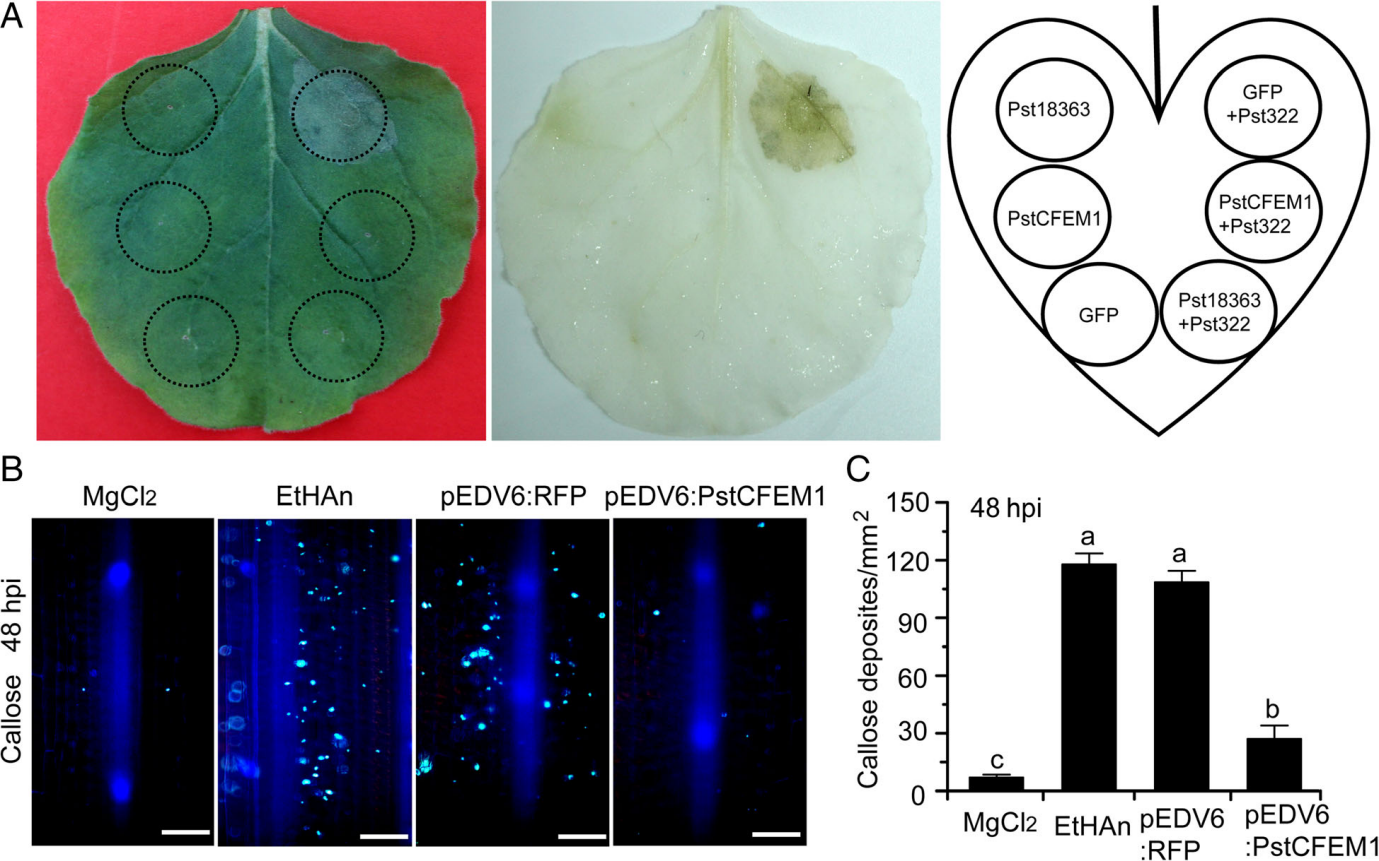
Overexpression of *PstCFEM1* suppressed programmed cell death and callose deposition. **A** PstCFEM1 suppressed Pst322-triggered cell death. *A. tumefaciens* cells respectively carrying the Pst18363, PstCFEM1 and eGFP vector were infiltrated into the leaf of *N. benthamiana* within the regions indicated by the dashed lines, followed after 24 h by either no further infiltration (left side) or infiltration with *A. tumefaciens* cells carrying the Pst322 (right side). The phenotype of cell death was scored and photographed at 5 d after infiltration with Pst322, and the leaves were decolorized with ethanol. **B** The wheat leaves inoculated with MgCl_2_ buffer, EtHAn, pEDV6:RFP and pEDV6:PstCFEM1 were examined for callose deposition by epifluorescence microscopy after aniline blue staining. Bar = 100 μm. **C** Average number of callose deposits in wheat leaves inoculated with MgCl_2_, EtHAn, pEDV6:RFP and pEDV6:PstCFEM1. Data represent the mean of three biological replicates. Different letters indicate significant differences (one-way ANOVA, Tukey’s Multiple Comparison Test, *p* < 0.05)

To explore the mechanisms by which PstCFEM1 contributes to pathogenicity, we investigated whether, as other fungal effectors, PstCFEM1 could suppress PCD in *N. benthamiana*. In this study, we chose the *Pst* candidate elicitor-like protein Pst322 as a trigger for cell death (Wang et al., 2012) and Pst18363 was used as a positive control to suppress PCD (Yang et al., 2020). The leaves were infiltrated with *A. tumefaciens* cells containing PstCFEM1, Pst18363 and GFP, then *A. tumefaciens* cells carrying Pst322 were infiltrated in the same position after 24 h. After 5 d of the final infiltration, no cell death symptoms were observed on the leaf pre-infiltrated with *A. tumefaciens* carrying PstCFEM1 and Pst18363, whereas Pst322-induced cell death symptoms were evident in regions pre-infiltrated with *A. tumefaciens* carrying GFP (Fig. 3A). As expected, *A. tumefaciens* carrying PstCFEM1 and GFP alone did not elicit cell death (Fig. 3A). The results indicated that overexpression of *PstCFEM1* suppressed Pst322-trigged cell death. We further investigated the function of PstCFEM1 in suppressing plant immunity by using the same procedure in *N. benthamiana*. After 24 h of the final infiltration, we observed a lower level of callose deposition in PstCFEM1 and Pst322 infiltrated leaves compared to the tobacco leaves with GFP and Pst322 infiltrated leaves (Fig. S6A). In addition, RT-PCR and western blot assays confirmed the normal expression of genes (*GFP*, *Pst18363*, *PstCFEM1* and *Pst322*) in the infiltrated regions (Fig. S6B, C).

It has been reported that the effector detector vector (EDV) can deliver individual non-bacterial effectors to host plant cells via the bacterial TTSS (Sohn et al., 2007). Therefore, *PstCFEM1* was also cloned into the expression vector pEDV6, and then delivered into wheat cv. Suwon11 by the modified *P. fluorescens* strain EtHAn, which carries a functional TTSS (Thomas et al., 2009; Yin & Hulbert, 2011). Callose deposition was observed on wheat leaves after inoculating with EtHAn (Fig. 3B), and the EtHAn strain-carrying RFP was used as the negative control. We observed a decrease in callose deposition in pEDV6:PstCFEM1-inoculated wheat plants (Fig. 3B). Next, we measured the callose deposition in the infiltrated wheat leaves and found that callose deposition in PstCFEM1-infiltrated leaves significantly decreased compared to that in leaves infiltrated with the EtHAn strain carrying pEDV6:RFP (Fig. 3C). These results indicated that overexpression of *PstCFEM1* suppresses PCD and callose deposition, and influences the virulence of PstCFEM1.

### Silencing of *PstCFEM1* weakens the virulence of *Pst*

**Fig. 4:**
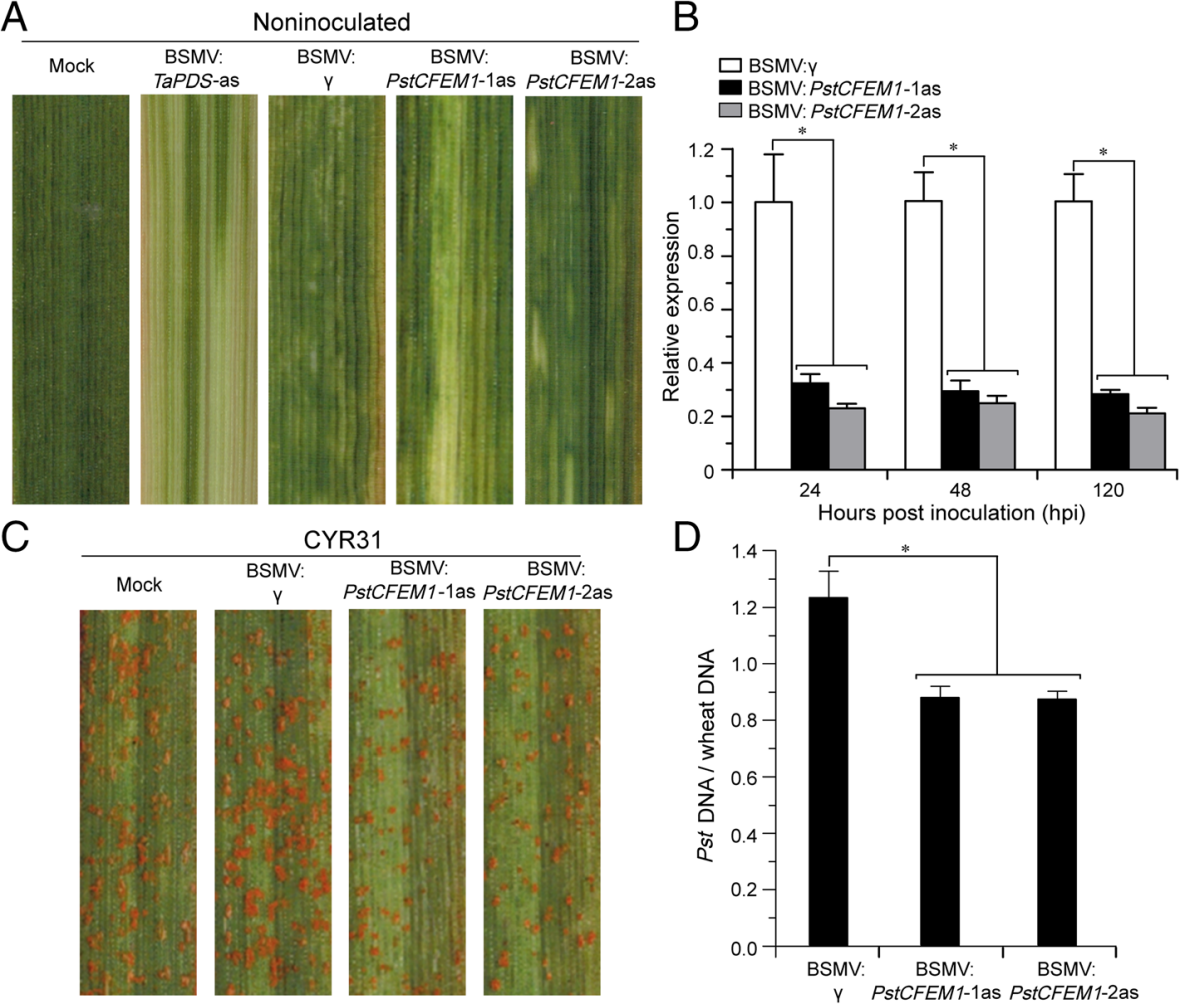
BSMV-mediated HIGS of *PstCFEM1* weaken the pathogenicity of *Pst*. **A** Mild chlorotic mosaic symptoms were observed on leaves inoculated with BSMV:γ, BSMV:*TaPDS*-as and BSMV:*PstCFEM1*–1as/2as at 10 dpi. Mock, wheat leaves treated with 1 × Fes buffer. **B** Relative expression of *PstCFEM1* during the interaction between wheat containing the *PstCFEM1*-silenced construct and CYR31. qRT-PCR values were normalized using *PstEF1*, and were presented as fold changes relative to that in plants with BSMV:γ treatment at time 0. **C** Photos of fourth leaves of wheat containing the *PstCFEM1*-silenced construct inoculated with fresh urediospores of the CYR31 race. Typical leaves were photographed at 14 dpi. **D** Ratio of fungal to wheat nuclear content determined using the contents of fungal *PstEF1* and wheat *TaEF-1α* genes, respectively. Genomic DNA was extracted from three different plants at 7 dpi. Data represent the mean of three biological replicates. Asterisks indicate significant differences between that in silenced plants and control plants at the same time points using student’s *t*-test (*, *P* < 0.05)

The HIGS technique has facilitated research on *Pst* effectors (Qi et al., 2019a; Xu et al., 2019). In this study, HIGS mediated by *Barley Stripe Mosaic Virus* (BSMV) was used to knock down the transcripts of *PstCFEM1*. Two fragments were selected in the coding region to specifically silence *PstCFEM1*. The second leaves of Suwon11 seedlings were inoculated with BSMV:*TaPDS* (wheat phytoene desaturase gene)-as, BSMV:*PstCFEM1*–1as/2as antisense (*PstCFEM1*-silenced) and BSMV:γ (control). A similar number of seedlings were also mock-inoculated with a buffer lacking BSMV. The photo-bleaching phenotype was observed when *TaPDS* was silenced (Fig. 4A). All wheat plants containing the *PstCFEM1*-silenced construct and control plants expressed mild chlorotic mosaic symptoms, confirming that the BSMV-mediated gene silencing system functioned correctly and could be used in further experiments (Fig. 4A). To clarify whether *PstCFEM1* was successfully silenced, transcript levels of *PstCFEM1* were examined by qRT-PCR assay, which indicated that its transcript was noticeably decreased at 24, 48, and 120 hpi (Fig. 4B). The fourth leaves of wheat plants were then inoculated with the virulent *Pst* isolate CYR31. The phenotypes showed that wheat plants containing the *PstCFEM1*-silenced construct had reduced sporulation (Fig. 4C) and they had significantly reduced biomass (Fig. 4D) compared to control plants.

**Fig. 5:**
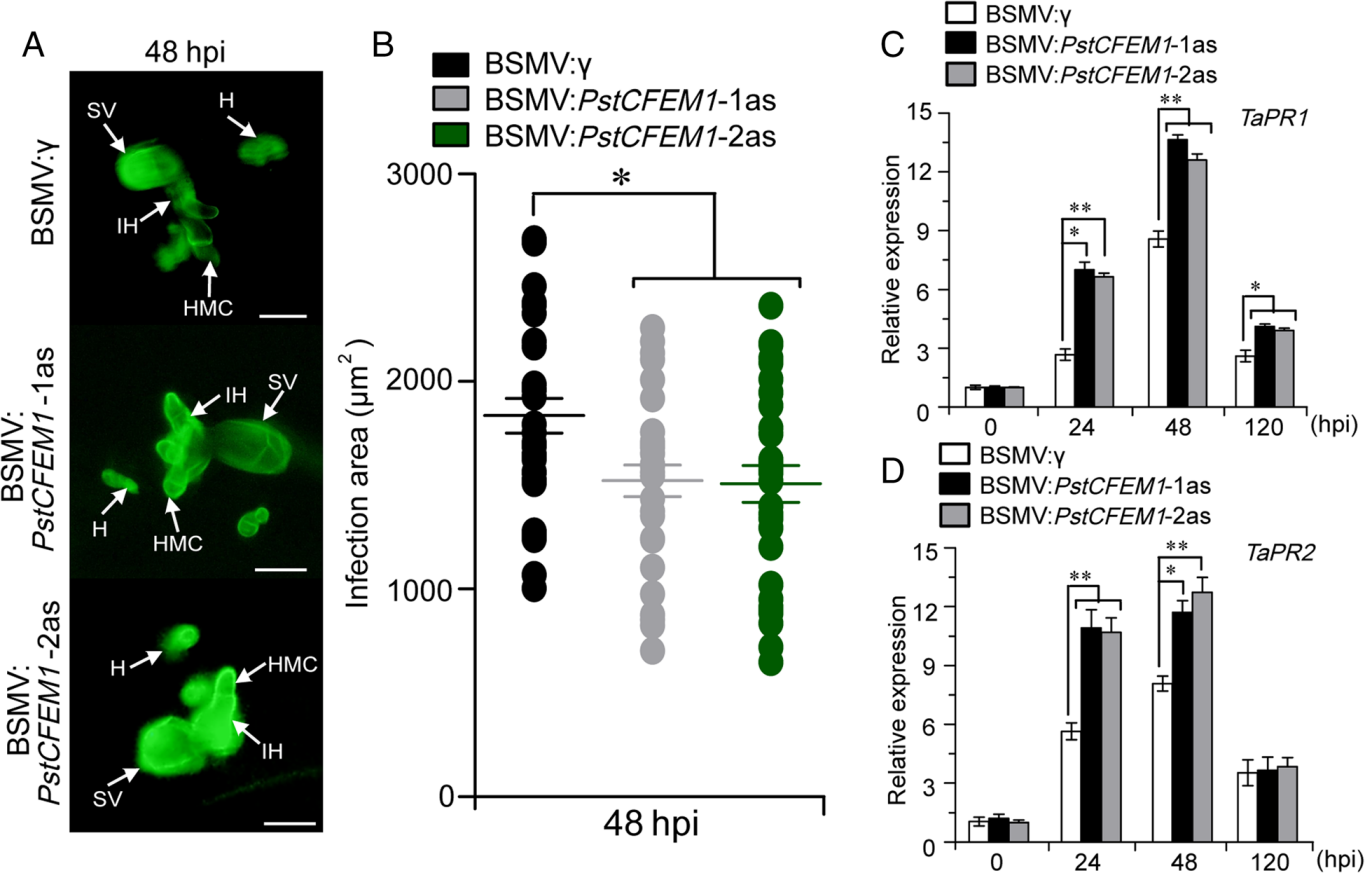
Histological observation of fungal growth and transcript levels of *TaPR* genes in wheat containing the *PstCFEM1*-silenced construct compared to control plant. **A** The fungal structures were stained with WGA in wheat leaves inoculated with *Pst* and observed under a fluorescence microscope. SV, sub-stomatal vesicle; HMC, haustorial mother cell; IH, infection hypha; H, haustoria. **B** The Infection area were calculated using DP-BSW software. All results were calculated from 30 to 40 infection sites. Transcript levels of (**C**) *TaPR1* and (**D**) *TaPR2* in *PstCFEM1*-silenced compared to control plants. qRT-PCR values were normalized with *TaEF-1α*. Error bar represents the variation among three independent replicates. All experiments were performed with three biological replicates. Asterisks indicate significant differences between that in silenced plants and control at the same time points using student’s *t*-test (*, *P* < 0.05; **, *P* < 0.01)

Based on the phenotypic variation of wheat containing the *PstCFEM1*-silenced construct inoculated with *Pst*, we assessed the growth of *Pst*. At 48 hpi, the colony area per infection site was obviously smaller in wheat containing the *PstCFEM1*-silenced construct compared with control plants (Fig. 5A, B), indicating that *PstCFEM1* impairs *Pst* growth. To further confirm the suppressed *Pst* growth in wheat containing the *PstCFEM1*-silenced construct, the transcripts of pathogenesis-related (*PR*) marker genes (*TaPR1* and *TaPR2*) (Liu et al., 2019), were examined by qRT-PCR assay. The transcript levels of *TaPR1* and *TaPR2* were significantly induced in wheat containing the *PstCFEM1*-silenced construct during *Pst* infection (Fig. 5C, D).

### Silencing of *PstCFEM1* increases ROS accumulation during *Pst*-wheat interaction

**Fig. 6:**
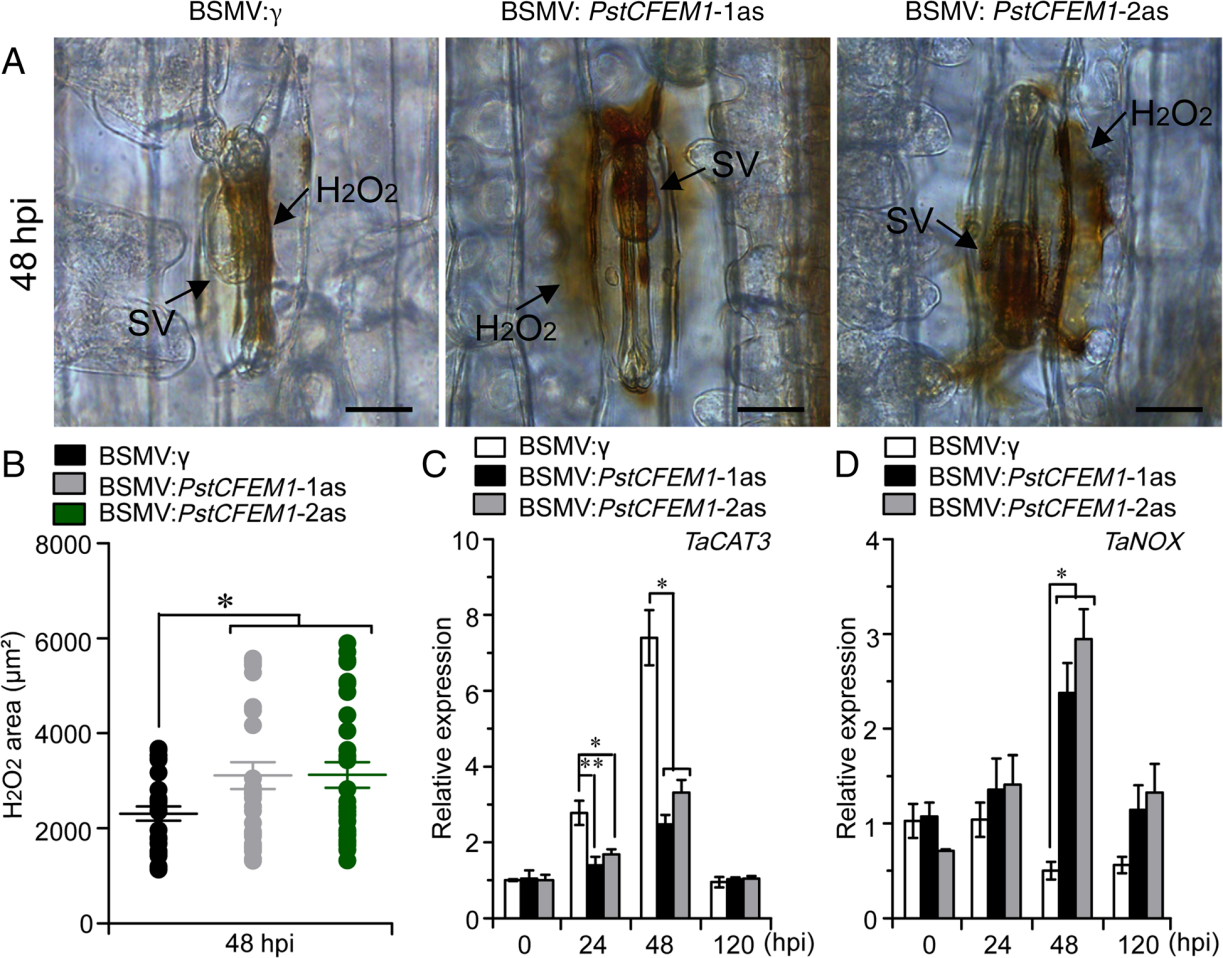
Production of ROS in control and wheat containing the *PstCFEM1*-silenced construct challenged with CYR31. **A** H_2_O_2_ production was observed by DAB staining. H_2_O_2_ accumulation and fungal growth in BSMV-silenced leaves at 48 hpi. Bars = 20 μm. SV, sub-stomatal vesicle; HMC, haustorial mother cell; IH, infection hypha and H, haustorium. **B** The H_2_O_2_ areas were calculated using DP-BSW software. All results were calculated from 30 to 40 infection sites. Transcript levels of (**C**) *TaCAT3* and (**D**) *TaNOX* in *PstCFEM1*-silenced compared to control plants. qRT-PCR values were normalized for *TaEF-1α*. Error bar represents the variation among three independent replicates. All experiments were performed with three biological replicates. Asterisks indicate significant differences between that in silenced plants and control plants at the same time points using student’s *t*-test (*, *P* < 0.05; **, *P* < 0.01)

To examine the host responses in wheat containing the *PstCFEM1*-silenced construct, we measured ROS accumulation by staining the infected wheat tissues at 48 hpi with DAB. H_2_O_2_ area per infection site in wheat containing the *PstCFEM1*-silenced construct was significantly increased compared with control plants (Fig. 6A, B). Meanwhile, the transcripts of two ROS-related marker genes (*TaCAT3* and *TaNOX*) (Hawku et al., 2021; Liu et al., 2019), were examined by qRT-PCR assay. *TaCAT3* was significantly decreased at 24 and 48 hpi in wheat containing the *PstCFEM1*-silenced construct compared with control plants (Fig. 6C), *TaNOX* exhibited a significantly increased transcript at 48 hpi in wheat containing the *PstCFEM1*-silenced construct compared with control plants (Fig. 6D). These results suggest that silencing of *PstCFEM1* induces host ROS accumulation.

### *PstCFEM1* inhibits ROS accumulation in *N. benthamiana*

**Fig. 7:**
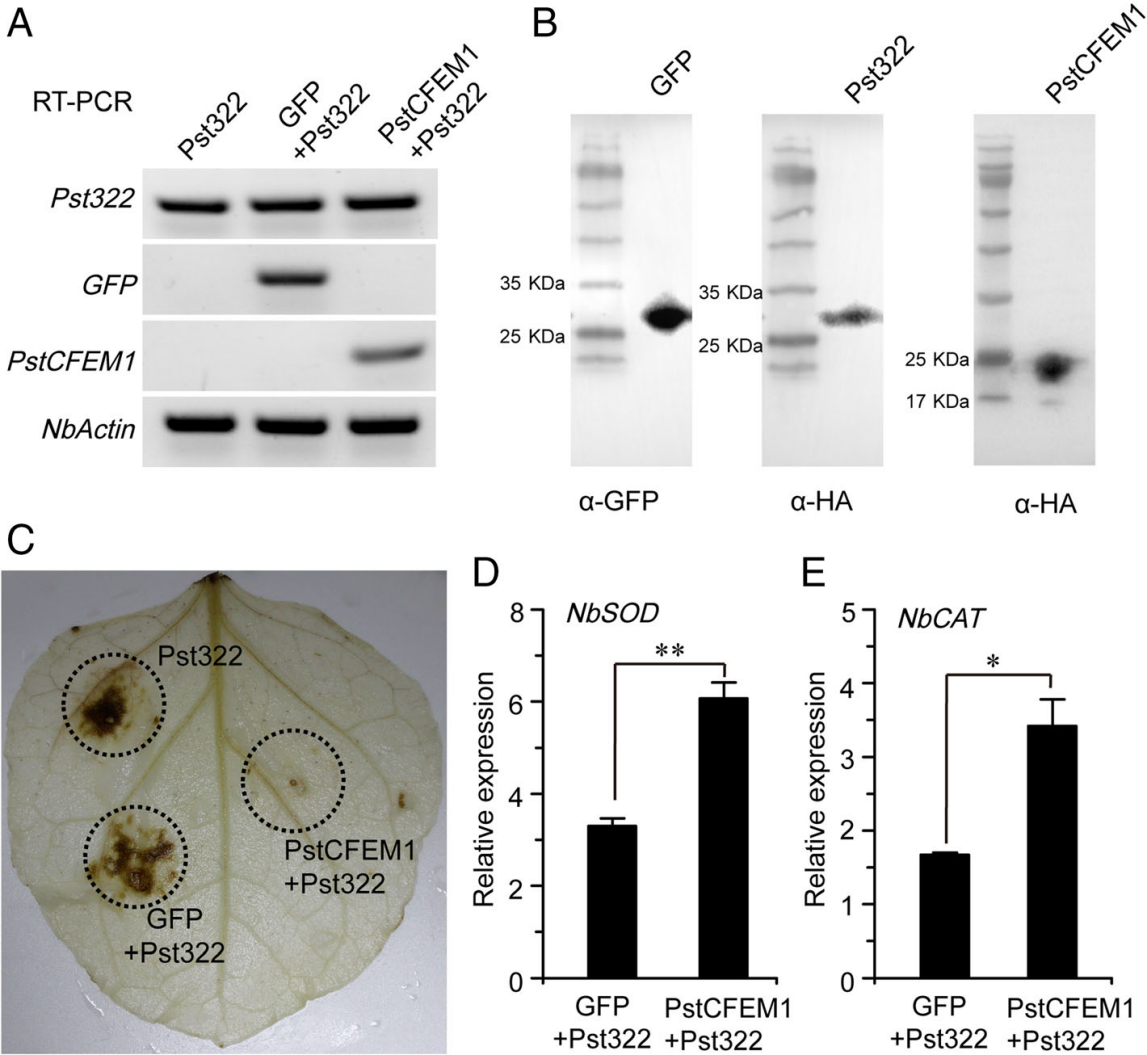
*PstCFEM1* decreases ROS accumulation. RT-PCR (**A**) and western blot (**B**) was performed to confirm the expression of PstCFEM1, GFP and Pst322 in *N. benthamiana*. *Nbactin* was used as a reference gene. **C** H_2_O_2_ production in *N. benthamiana* leaves was determined by DAB staining. *N. benthamiana* leaves were infiltrated with *A. tumefaciens* cells carrying the PstCFEM1 or GFP, followed after 24 h by infiltration with *A. tumefaciens* cells carrying the Pst322. The measurement was performed 2 d after infiltration with Pst322. Transcript levels of (**D**) *NbSOD* and (**E**) *NbCAT* in PstCFEM1 + Pst322 region compared to GFP + Pst322 region at 1d after infiltration with Pst322. qRT-PCR values were normalized with *Nbactin*. Error bar represents the variation among three independent replicates. All experiments were performed with three biological replicates. Differences between PstCFEM1 + Pst322 region and GFP + Pst322 region were assessed using student’s *t*-test (*, *P* < 0.05; **, *P* < 0.01)

Because ROS is a crucial trigger of cell death and to test if PstCFEM1 suppresses Pst322-triggered cell death by preventing ROS accumulation, DAB staining was used to examine the ROS levels in infiltrated leaves of *N. benthamiana*. RT-PCR and western blot assays confirmed the normal expression of genes (*GFP*, *PstCFEM1* and *Pst322*) in the infiltrated regions (Fig. 7A, B). The DAB staining in the leaf regions infiltrated with PstCFEM1/Pst322 was much weaker compared with those infiltrated with buffer/Pst322 and GFP/Pst322 (Fig. 7C). Meanwhile, we used qRT-PCR assay to test the transcript levels of two ROS-scavenging genes (*NbSOD* and *NbCAT*) (Guo et al., 2018). *NbSOD* (Fig. 7D) and *NbCAT* (Fig. 7E) exhibited a significantly increased transcript level at 1 d in the PstCFEM1/Pst322 region compared with the GFP/Pst322 region. These results indicated that PstCFEM1 suppresses Pst322-triggered cell death by preventing ROS accumulation.

### Wheat plants containing the *PstCFEM1*-silenced construct express broad-spectrum resistance to *Pst*

**Fig. 8:**
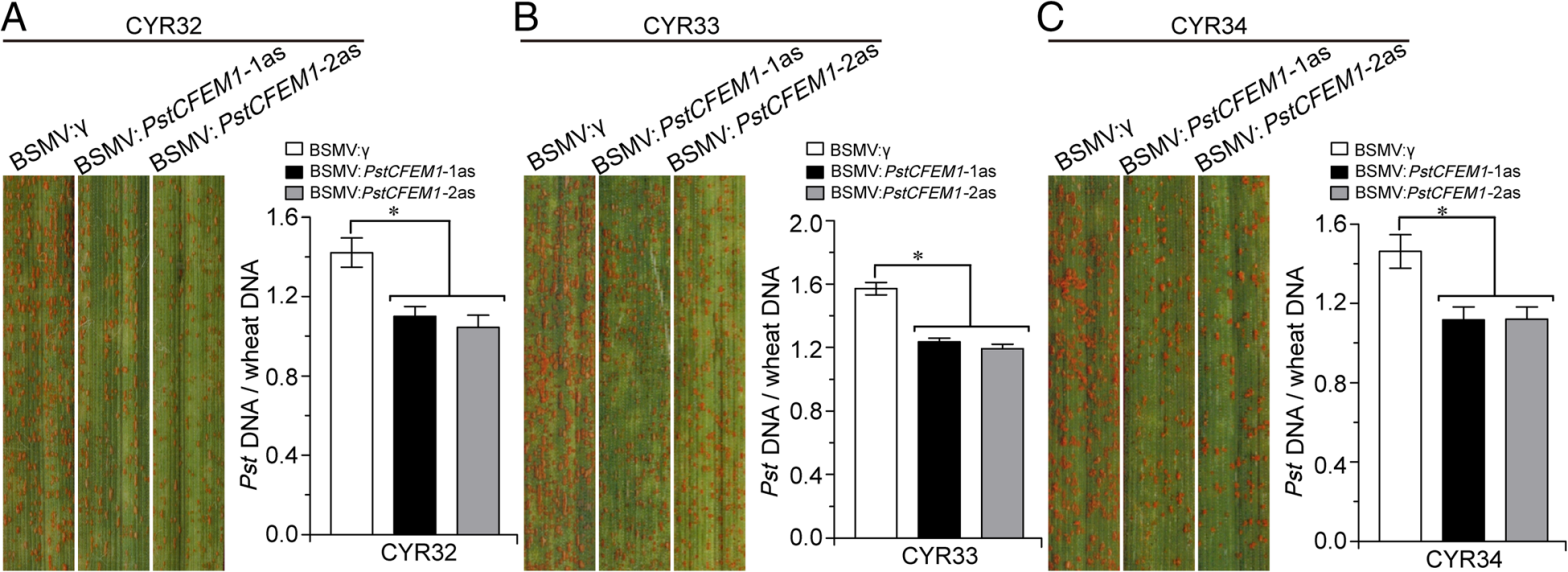
*PstCFEM1*-silenced plants expressed broad-spectrum resistance to *Pst*. Foliar parts of BSMV:*PstCFEM1*–1as/2as plants inoculated with *Pst* isolate CYR32 (**A**), CYR33 (**B**), and CYR34 (**C**). Ratio of fungal to wheat nuclear DNA content was determined using the contents of fungal *PstEF1* and wheat *TaEF-1α* genes, respectively. Genomic DNA was extracted from three different plants at 7 days post inoculation. Error bars represent the variation among three independent replicates. Asterisks indicate significant differences between that in silenced plants and control plants using student’s *t*-test (*, *P* < 0.05)

HIGS employing RNA silencing mechanism and, specifically, silencing the targets of invading pathogens, has been successfully applied in crop disease prevention (Qi et al., 2019b). Because the current prevalent *Pst* races in China are CYR32, CYR33, and CYR34 (Liu et al., 2017), we cloned the *PstCFEM1* gene in those three races and found by sequence analysis that *PstCFEM1* is conserved in different races (Fig. S7). We used the same method above to silence *PstCFEM1*, and found that both wheat plants containing the *PstCFEM1*-silenced construct and control plants were susceptible to CYR32 (Fig. 8A), CYR33 (Fig. 8B), and CYR34 (Fig. 8C), Compared with control plants, fungal biomass was significantly reduced in wheat containing the *PstCFEM1*-silenced construct (Fig. 8A-C). Thus, our results indicate that silencing of *PstCFEM1* confers broad-spectrum resistance of wheat to *Pst*.

## Discussion

CFEM domains are unique to fungi and originated from the common ancestor of *Ascomycota* and *Basidiomycota* (Zhang et al., 2015). The original function of CFEM domains is cell wall/membrane constitution, but divergence from normal functioning facilitates other various roles, such as pathogenicity (Zhang et al., 2015). MoPTH11 is an important G-protein-coupled receptor (GPCR) containing seven transmembrane domains (Kou et al., 2016; Weis et al., 2018), and the CFEM protein CSA1 of *Candida albicans* and AG2 of *Coccidioides immitis* also are membrane proteins (Peng et al., 1999; Lamarre et al., 2000). CFEM proteins without transmembrane structure usually comprise GPI-anchored sites, such as CFEM-domain GPI-anchored proteins (CfmA-C) and BcCFEM1 (Vaknin et al., 2014; Zhu et al., 2017). In plants, GPI-anchored proteins are involved in regulation of cell expansion and cell wall biosynthesis (Brady et al., 2007). Several studies have indicated that GPI-anchored proteins attach to the plasma membrane and play an important role in maintaining the cell wall and stress tolerance (Kitagaki et al., 2002). In this study, we found that PstCFEM1 has a predicted GPI-anchor site (Fig. S4), and it’s an apoplastic effector (Fig. S5; Fig. 2B). Apoplastic effectors play roles in extracellular spaces and cope with physical and chemical barriers to break the first line of plant defenses (Giraldo et al., 2013; Tariqjaveed et al., 2021). Meanwhile, PstCFEM1 was proven to be a virulence effector in this study. Therefore, we propose that PstCFEM1 is a potential GPI-anchored CFEM protein, which is anchored to the outer layer of the cell membrane through a C-terminal GPI anchor, and it’s an apoplastic effector in the host apoplast during *Pst*-wheat interaction.

The CFEM domain has been reported to regulate mycelial growth, appressorium formation and pathogenicity (Salomon et al., 2014; Kou et al., 2016), but some CFEM proteins are avirulence factors or perform other functions. The three *Aspergillus fumigatus* CFEM-domain GPI-anchored proteins affect cell-wall stability but do not perform a role in fungal virulence (Vaknin et al., 2014). MoCDIP2 in the rice blast fungus is the only CFEM effector that can induce PCD (Chen et al., 2013), and we speculate that it may act as an elicitor-like protein. In *Fusarium graminearum,* none of the CFEM-containing GPCRs is essential for infection (Jiang et al., 2019). This indicates that functions of CFEM proteins may be diverse. In this study, we identified a CFEM domain protein PstCFEM1 from *Pst* that suppresses Pst322-induced cell death (Fig. 3A). Through transient overexpression in wheat, we found that it can inhibit callose accumulation (Fig. 3B, C), suggesting that PstCFEM1 is a virulence effector. In addition, a previous study reported that CFEM protein possesses antioxidant properties in *M. oryzae* (Kou et al., 2017). A similar result, that PstCFEM1 can affect ROS accumulation in host plants, was also observed in this study (Fig. 6, 7).

Pathogens secrete effectors into host cells and manipulate the host to promote their infection and colonization (Presti et al., 2015). At present, a variety of effectors have been known in different rust fungus. AvrM, AvrL567, Avr123 and AvrP4 have been reported in *Uromyces striatus* (Kemen et al., 2005), and the transfer protein RTP was identified in *Uromyces fabae* (Kemen et al., 2013; Pretsch et al., 2013). Pst_8713, Pst18363, PstGSRE1, Pst_12806 and PEC6 were identified in *Ps*t (Liu et al., 2016; Zhao et al., 2018; Xu et al., 2019; Qi et al., 2019a; Yang et al., 2020). These identified effectors are secreted proteins with unknown functions. In this study, we found a CFEM-containing protein in *Pst* that is a virulence effector suppressing accumulation of ROS in wheat and promoting the growth of *Pst*. Because the length and architecture of CFEM domains are relatively conserved (Zhang et al., 2015), we speculate that the orthology of PstCFEM1 in other rust fungi also has a similar function. In addition, *Pst* is a biotrophic, obligate parasite. It invades the host cell through the haustorium, which contributes in the exchange of nutrition and signals (Rafiqi et al., 2012). Moreover, because rust fungi cannot be cultured in vitro and cannot be genetically transformed, their study is relatively lagging (Voegele & Mendgen, 2011). Bacterial TTSS-mediated overexpression and HIGS technology have provided effective methods for the identification and functional research of rust effectors (Zhao et al., 2018; Qi et al., 2019b). In summary, our study provides novel insights into CFEM-containing proteins in host-pathogen interactions.

## Conclusion

In summary, our study revealed that PstCFEM1 suppresses wheat defense by inhibiting ROS accumulation and contributes to pathogenicity of *Pst*. In addition, wheat plants containing the *PstCFEM1*-silenced construct showed increased resistance to multiple races of *Pst*. This is the first evidence indicating that silencing a vital CFEM domain-containing protein gene confers broad-spectrum resistance to wheat stripe rust.

## Methods

### Plant materials and fungal isolates

Wheat cultivar Suwon11 was used for gene transcription analysis and HIGS assays (Liu et al., 2019). This cultivar, carrying the *YrSu* gene, is highly susceptible to CYR31 (Cao et al., 2003). Wheat seedlings were inoculated with *Pst* and maintained according to the procedures and conditions previously described (Wang et al., 2007). For RNA extraction, the second leaves inoculated with CYR31 or treated with sterile distilled water (control) were harvested at 0, 6, 12, 18, 24, 48, 72, and 120 hpi. *Pst* races CYR31, CYR32, CYR33 and CYR34 were used in this study. Suwon11 showed compatible interaction with *Pst* races CYR31, CYR32, CYR33, and CYR34.

### RNA extraction and qRT-PCR assays

The Quick RNA Isolation Kit (Huayueyang Biotechnology, China, Beijing) was used to extract RNA from all samples. The HiScript® QRT SuperMix for qPCR (Vazyme, Nanjing, China) was used for reverse transcription with 2 mg of total RNA. qRT-PCR was performed on a CFX Connect Real-Time System (Bio-Rad, USA). Table S1 in the supporting information contains a list of the primers used. *PstEF1* and *TaEF-1α* were used to normalize values obtained with the qRT-PCR assay (Bai et al., 2021).

### Yeast signal sequence trap system

The yeast signal sequence trap method was used as previously described (Yin et al., 2018). Using specific primers, we cloned the predicted signal peptide sequence of *PstCFEM1* into vector pSUC2 (Table S1). Then it was transformed into YTK12, an invertase mutant yeast strain (Oh et al., 2009). Invertase enzymatic activity was detected by reduction of TTC to insoluble red formazon TPF according to the procedures and conditions previously described (Jacobs et al., 1997). The specific method is the same as used in our previous study (Yang et al., 2020).

### *Agrobacterium tumefaciens* infiltration assays

The suppression of BAX- or Pst322-triggered PCD by PstCFEM1 was tested with the *A. tumefaciens*-mediated transient expression system (Zhao et al., 2018). The PVX-PstCFEM1 plasmid was constructed by amplifying the sequence encoding PstCFEM1 minus the signal peptide with primers listed in Table S1. *N. benthamiana* leaves were infected with resuspended *Agrobacterium* carrying eGFP or Pst18363 or PstCFEM1 at a final OD600 of 0.2 and 10 mM MgCl_2_ buffer (Bos et al., 2006). The inoculation sites were infiltrated with *A. tumefaciens* carrying BAX or Pst322 at a final OD600 of 0.2 in the same position at 24 hpi. The infiltrated *N. benthamiana* plants were placed in a glasshouse at a temperature of 25 °C in high intensity light. Three days after infiltration, RT-PCR and western blot was used to detect gene expression in all infiltration sites. At 4–5 days after infiltration, symptoms were observed and recorded. For each experiment, three biological replicates were carried out independently.

The apoplastic fluid from *N. benthamiana* leaves was extracted by the infiltration-centrifugation method as described (Nie et al., 2019). We cloned the *PstCFEM1* sequence into vector pCAMBIA1302 using specific primers (Table S1) and *N. benthamiana* leaves were infected with resuspended *Agrobacterium* carrying empty vector or PstCFEM1-GFP or Pst18363-GFP at a final OD600 of 0.2 and 10 mM MgCl_2_ buffer. At 2–3 days after infiltration, the samples were extracted.

### Bacterial TTSS-mediated overexpression in wheat plants

Electroporation was used to introduce the pEDV6:PstCFEM1 construct into the *P. fluorescens* strain EtHAn. Bacterial cells expressing PstCFEM1 were cultured overnight at 28 °C in KB medium (Yin & Hulbert, 2011), then collected and resuspended in an infiltration medium for transient expression in wheat cells (10 mM MgCl_2_). The *P. fluorescens* strain EtHAn was diluted to an OD600 of 0.6 and infiltrated using a syringe without a needle into the second leaves of wheat cultivar Suwon11. The infiltrated wheat plants were maintained at a constant temperature of 25 °C in a growth chamber. The wild-type strain EtHAn was used to trigger PTI. As a negative control, wheat leaves were infiltrated with recombinant EtHAn carrying the RFP. Leaf samples were stained with aniline blue to analyze the suppression of callose deposition as previously described (Hood & Shew, 1996). An Olympus BX-51 microscope was used to analyze the specimens (Olympus, Japan). All experiments were repeated three times.

### BSMV-mediated *PstCFEM1* gene silencing

Two specific cDNA fragments of *PstCFEM1* were obtained for HIGS analysis, we confirmed that the fragments showed nonsimilarity with any other genes by Si-Fi software analysis of the wheat and *Pst* databases. BSMV constructs (BSMV:*PstCFEM1*–1as / 2as) were used to inoculate wheat seedlings. The control for the BSMV infection test was BSMV:*TaPDS*, which contained the wheat phytoene desaturase (PDS) gene. Mock inoculations were performed with 1 × FES buffer (Yang et al., 2020). At 9–12 days after virus inoculation, the fourth leaves were inoculated with fresh urediniospores. At 14–16 days after *Pst* inoculation, the phenotypes of the fourth leaves were examined and photographed, and the leaves were collected for RNA extraction at 24, 48, and 120 hpi (Qi et al., 2018). The silencing efficiencies of *PstCFEM1* were assessed by qRT-PCR. Fungal biomass was measured as previously studied (Qi et al., 2018). *Pst* growth and H_2_O_2_ accumulation in wheat plants were analyzed using histological tools. H_2_O_2_ was detected using the DAB staining process. An Olympus BX-51 microscope was used to analyze the stained samples (Olympus, Japan). Decolorized leaf segments were stained with wheat germ agglutinin (WGA) conjugated to Alexa-488 (Invitrogen, USA). Three biological replicates were used in this assay.

## Supplementary Information

Additional file 1: **Supplementary Fig. 1.** PstCFEM1 in *Pst* suppresses plant cell death triggered by BAX in *N. benthamiana*.

Additional file 2: **Supplementary Fig. 2.** SignalP 5.0 predicts that PstCFEM1 has a signal peptide.

Additional file 3: **Supplementary Fig. 3.** TMHMM predicts that PstCFEM1 lack a transmembrane domain.

Additional file 4: **Supplementary Fig. 4.** GPI-anchor Predictor predicts that PstCFEM1 has a GPI-anchor site.

Additional file 5: **Supplementary Fig. 5.** EffectorP 3.0 predicts that PstCFEM1 is an apoplastic effector.

Additional file 6: **Supplementary Fig. 6. A** Overexpression of *PstCFEM1* suppressed Pst322-triggered callose deposition in *N. benthamiana*. **B** RT-PCR and (**C**) western blot were performed to confirm the expression of *Pst18363*, *PstCFEM1*, *GFP* and *Pst322* in each infiltration site with specific primers. *Nbactin* was used as a reference gene.

Additional file 7: **Supplementary Fig. 7.** Alignment of the *PstCFEM1* coding regions in CYR32, CYR33 and CYR34 races.

Additional file 8: **Supplementary Table 1.** Primers used in this study.

## Data Availability

All data and materials are available in the paper and online supplemental files.
