# Supplementary figures and images for: A candidate effector protein PstCFEM1 contributes to virulence of stripe rust fungus and impairs wheat immunity
Source: Stress Biol. 2022 Apr 8;2(1):21. doi: 10.1007/s44154-022-00042-5 (PMC10441960; doi:10.1007/s44154-022-00042-5)

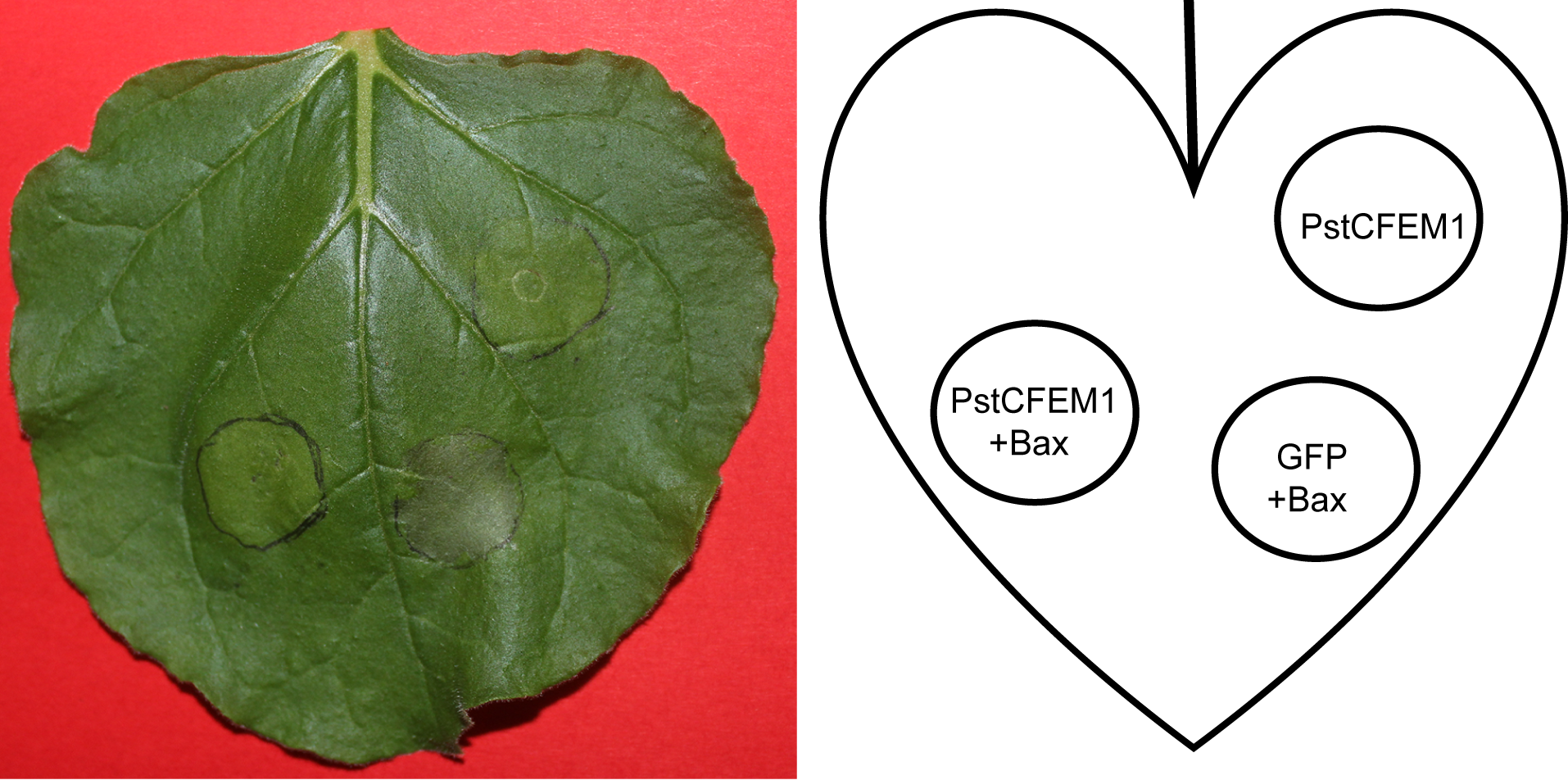

Supplement: Supplementary file 1 — Supplementary Fig. 1. PstCFEM1 in Pst suppresses plant cell death triggered by BAX in N. benthamiana. [file 44154_2022_42_MOESM1_ESM.tif]

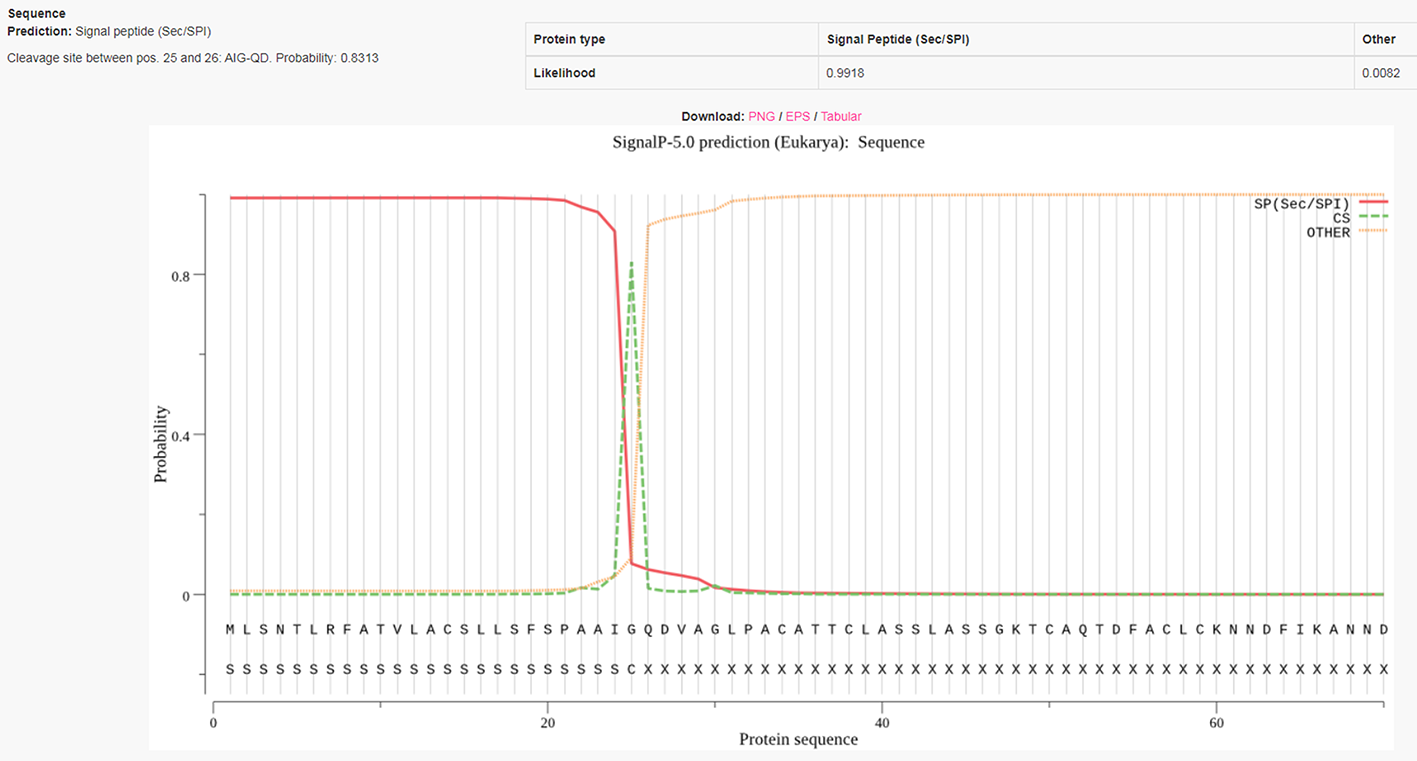

Supplement: Supplementary file 2 — Supplementary Fig. 2. SignalP 5.0 predicts that PstCFEM1 has a signal peptide. [file 44154_2022_42_MOESM2_ESM.tif]

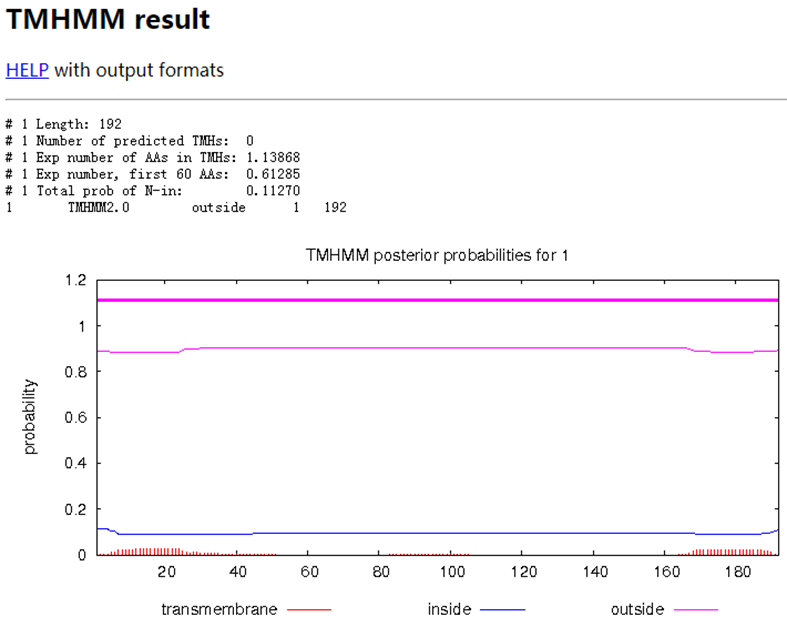

Supplement: Supplementary file 3 — Supplementary Fig. 3. TMHMM predicts that PstCFEM1 lack a transmembrane domain. [file 44154_2022_42_MOESM3_ESM.tif]

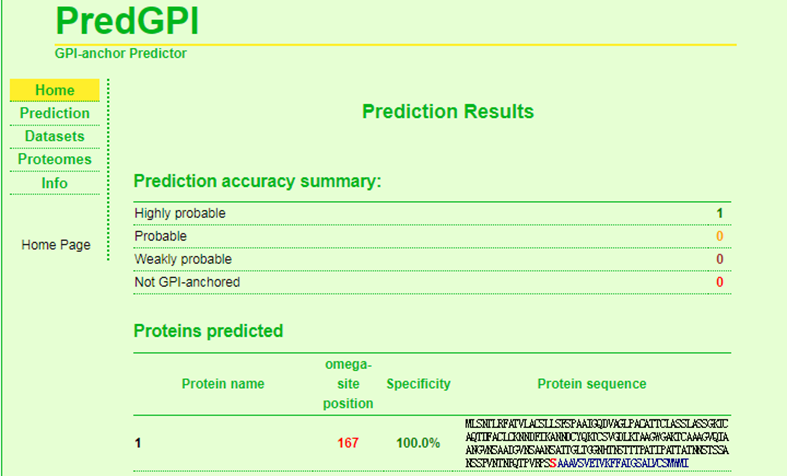

Supplement: Supplementary file 4 — Supplementary Fig. 4. GPI-anchor Predictor predicts that PstCFEM1 has a GPI-anchor site. [file 44154_2022_42_MOESM4_ESM.tif]

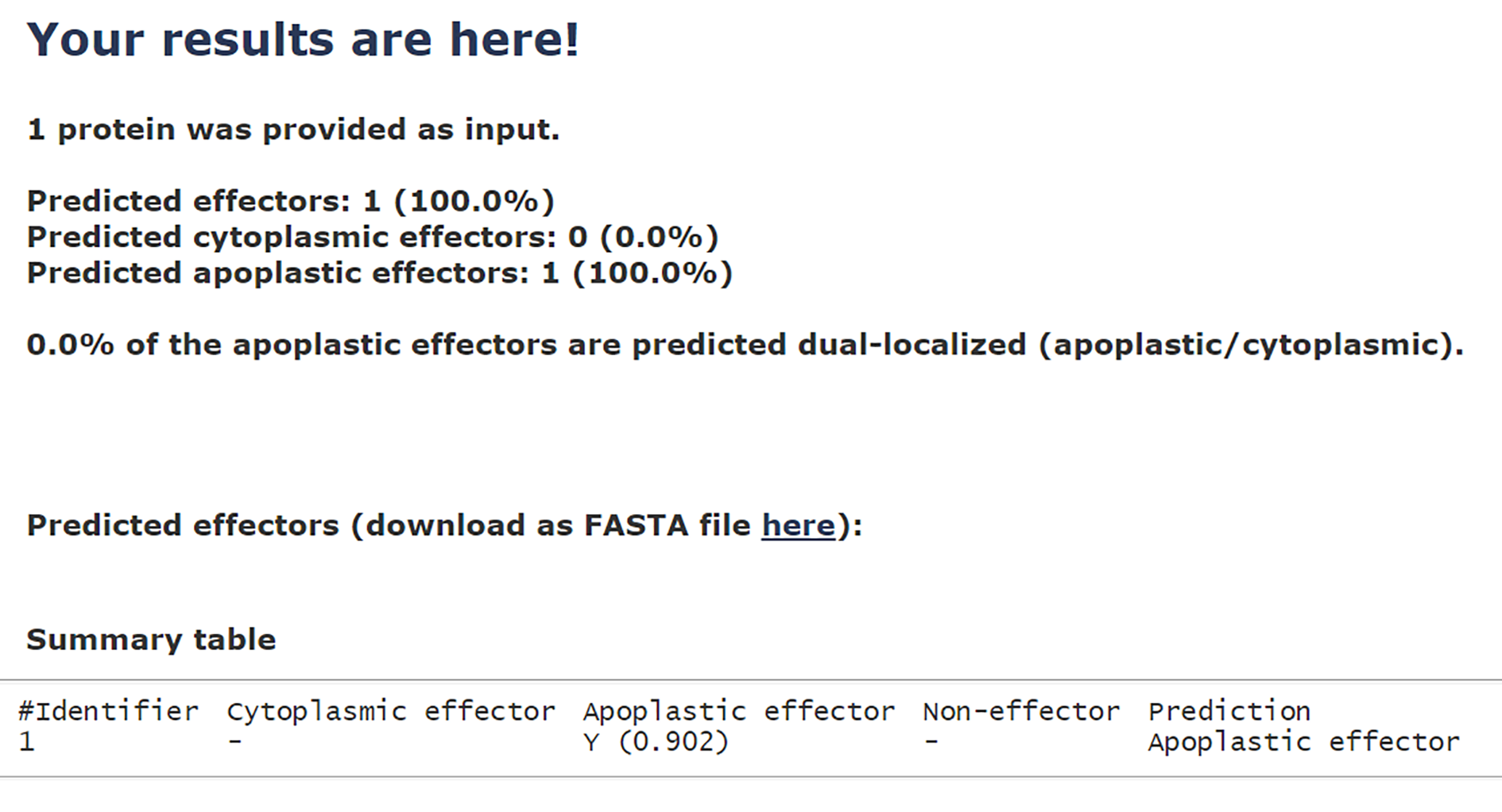

Supplement: Supplementary file 5 — Supplementary Fig. 5. EffectorP 3.0 predicts that PstCFEM1 is an apoplastic effector. [file 44154_2022_42_MOESM5_ESM.tif]

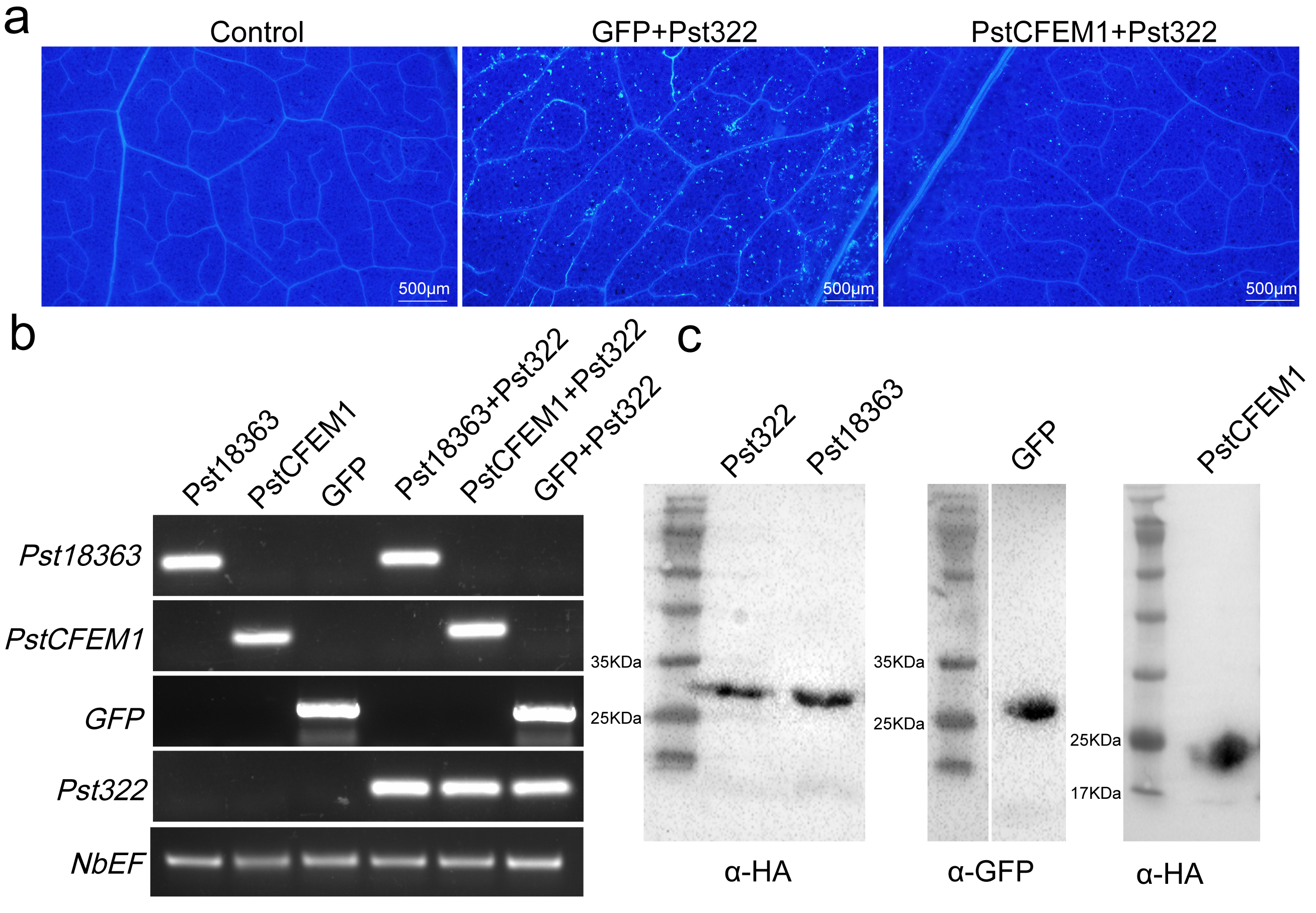

Supplement: Supplementary file 6 — Supplementary Fig. 6. A Overexpression of PstCFEM1 suppressed Pst322-triggered callose deposition in N. benthamiana. B RT-PCR and (C) western blot were performed to confirm the expression of Pst18363, PstCFEM1, GFP and Pst322 in each infiltration site with specific primers. Nbactin was used as a reference gene. [file 44154_2022_42_MOESM6_ESM.tif]

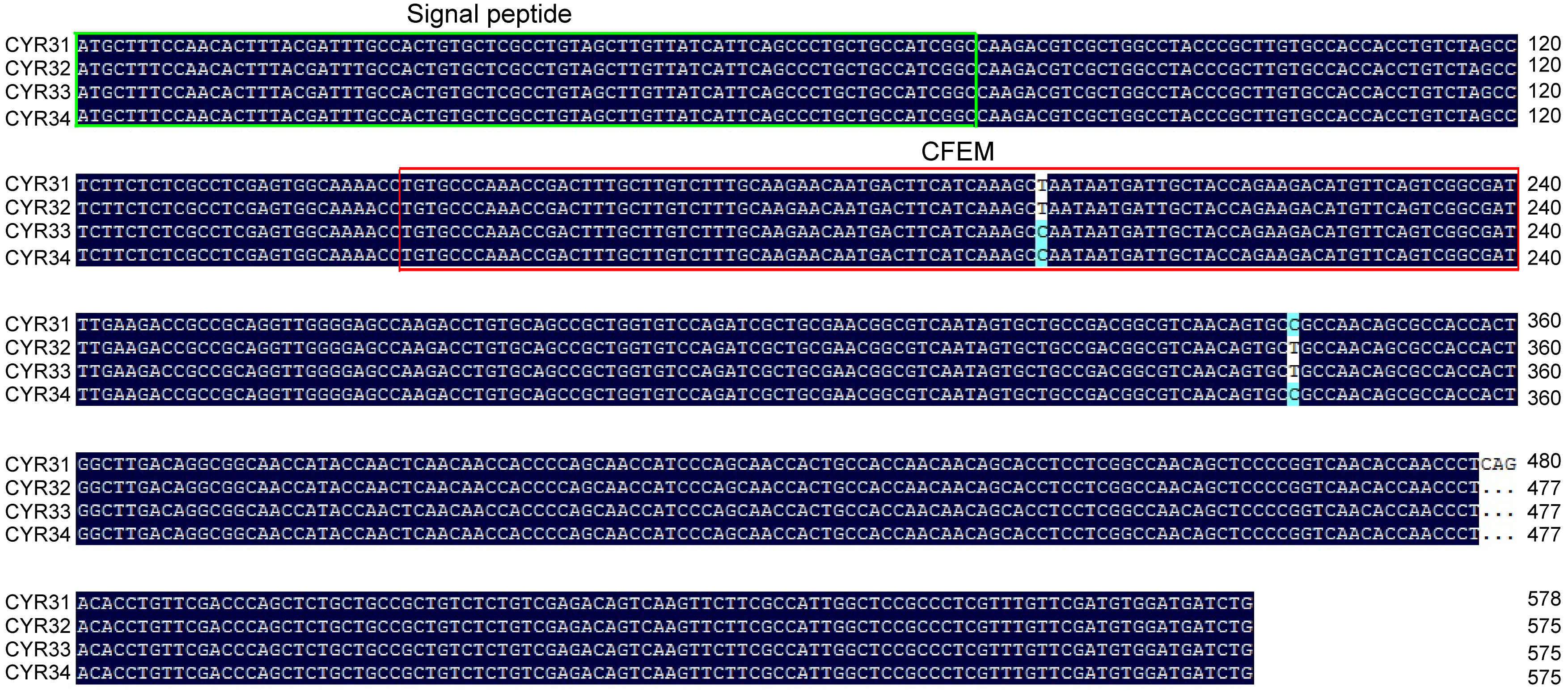

Supplement: Supplementary file 7 — Supplementary Fig. 7. Alignment of the PstCFEM1 coding regions in CYR32, CYR33 and CYR34 races. [file 44154_2022_42_MOESM7_ESM.tif]
